# Contributions of the MyD88-Dependent Receptors IL-18R, IL-1R, and TLR9 to Host Defenses following Pulmonary Challenge with *Cryptococcus neoformans*

**DOI:** 10.1371/journal.pone.0026232

**Published:** 2011-10-19

**Authors:** Jennifer P. Wang, Chrono K. Lee, Ali Akalin, Robert W. Finberg, Stuart M. Levitz

**Affiliations:** Department of Medicine, University of Massachusetts Medical School, Worcester, Massachusetts, United States of America; University of Cincinnati - College of Medicine, United States of America

## Abstract

Signaling via the adapter protein, MyD88, is important in the host defense against *Cryptococcus neoformans* infection. While certain Toll-like receptors (TLRs) can enhance the clearance of Cryptococcus, the contributions of MyD88-dependent, TLR-independent pathways have not been fully investigated. We examined the roles of IL-1R and IL-18R in vivo by challenging C57BL/6 mice with a lethal strain of Cryptococcus. We found that the absence of IL-18R, but not IL-1R, causes a shift in the survival curve following pulmonary delivery of a virulent strain of *C. neoformans* (H99). Specifically, IL-18R-deficient mice have significantly shorter median survival times compared to wild-type mice following infection. Cytokine analysis of lung homogenates revealed that deficiency of IL-IR, IL-18R, or MyD88 is associated with diminished lung levels of IL-1β. In order to compare these findings with those related to TLR-deficiency, we studied the effects of TLR9-deficiency and found that deficiency of TLR9 also affects the survival curve of mice following challenge with *C. neoformans.* Yet the lungs from infected TLR9-deficient mice have robust levels of IL-1β. In summary, we found that multiple signaling components can contribute the MyD88-dependent host responses to cryptococcal infection in vivo and each drives distinct pulmonary responses.

## Introduction

*Cryptococcus neoformans* is a fungal pathogen that causes life-threatening disease preferentially in individuals with impaired T cell function, particularly persons with AIDS [1]. The route of infection is typically through inhalation into the lung. In most patients, dissemination into the central nervous system is observed. Globally, it is estimated that approximately one million people per year acquire cryptococcal meningitis with over 600,000 attributable deaths [2].

Investigations of the host responses to *C. neoformans* using murine models of infection have broadened our understanding of the signaling pathways involved in defense. Toll-like receptors (TLRs) are innate immune receptors that are critical in the host defense against many types of invading pathogens. Most TLRs utilize the adaptor molecule MyD88 for signaling. Following activation via TLRs and MyD88, innate immune cells produce cytokines and effector molecules essential for the adaptive immune response. The importance of MyD88 in host defense in cryptococcal infection was demonstrated when MyD88-deficient mice exhibited decreased survival and increased fungal burden in the lungs following fungal challenge [3], [4]. In addition, a limited protective role for TLR2, but none for TLR4, was found in these studies [3], [4]. Recent investigations have focused on the role of TLR9 in cryptococcal infection. Namakura et al. found that TLR9-deficient mice had an increased cryptococcal burden in lungs than wild-type (WT) mice and established that following stimulation with *C. neoformans*, bone marrow dendritic cells (BM-DC) from TLR9-deficient mice produced lower amounts of IL-12p40 than those from WT mice [5]. TLR9 has also been shown to contribute to clearance of *Cryptococcus* from lungs through recruitment of effector cells including macrophages and lymphocytes [6]. However, the impact of TLR9 on survival during cryptococcosis has not been described.

In addition to being utilized by the TLRs, the MyD88 adapter molecule is required for the IL-1 and IL-18 signaling pathways [7], [8]. IL-1β and IL-18 are structurally related cytokines that are important for the initiation of the inflammatory cascade. Both are synthesized as pro-peptides that are cleaved by active caspase-1 for cytokine maturation. While IL-18 has been reported to play a protective role in models of Cryptococcus infection [9], [10], the contribution of the IL-18 receptor (IL-18R) itself has not been specifically studied. Furthermore, published studies investigating the contributions of TLRs and IL-18 in mice infected with Cryptococcus use different fungal strains and outcome measures.

The goal of this study was to define the relative contributions of the IL-18R and IL-1R signaling pathways in the pathogenesis of *C. neoformans* infection in vivo.

Survival, fungal burden in lungs, brain, and spleen, and lung histopathology were compared between Cryptococcus-infected IL-1R-, IL-18R-, and MyD88-deficient mice and wild-type mice. We also examined chemokine and cytokine levels in lungs from these animals to gain insights into the pathway-specific induction. Finally, we examined TLR9 knockout mice using this same infection model to define its specific contribution towards MyD88-dependent pathogenesis in a unified manner.

## Results

### IL-18R and MyD88 both influence host survival to C. neoformans infection

We infected mice with a dose (2×10^4^ colony forming units, or CFU) of the highly virulent H99 strain of *C. neoformans* delivered by the intranasal (i.n.) route and monitored mice for survival for 50 days. Mice from all groups displayed evidence of disease including staggered gait, lethargy, diminished responsiveness, and bulging necks. Many mice lost the ability to right themselves. Typically 10–20% of WT mice survived 50 days post fungal challenge, the time at which the experiment was terminated. These surviving mice did not display any evidence of disease on gross inspection of lung and brain tissue.

**Figure 1:**
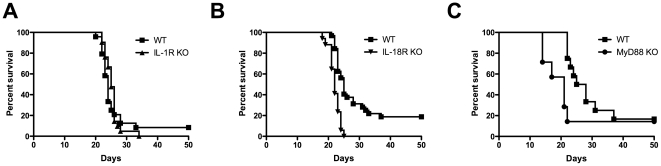
Survival of C57BL/6 wild-type, IL-18R knockout, IL-1R knockout, and MyD88 knockout mice following infection with *C. neoformans*. Mice were infected i.n. with 2×10^4^ *C. neoformans* organisms and were monitored for signs of disease and euthanized when the signs of disease were severe. (A) No difference in survival was observed following infection of wild-type (*n = 24*) and IL-1R knockout (*n = 21*) mice with *C. neoformans*. Data are combined from two independent experiments, each with similar results. (B) The survival curves were significantly different between IL-18R knockout mice (*n = 17*) and wild-type mice (*n = 32*), *P*<0.0001. The data are combined from three independent experiments, each of which had similar results. (C) MyD88 knockout mice (*n = 7*) had a trend towards diminished survival compared to wild-type (*n = 12*), *P* = 0.058.

IL-1R knockout mice did not exhibit any significant survival differences compared to WT mice (**Figure 1A**). The median survival time was 24 days for WT mice and 25 days for IL-1R deficient mice. In contrast, at this same dose, IL-18R knockout mice had a significant decrease in median survival time compared to WT mice following infection with *C. neoformans* strain H99 (**Figure 1B**). The median survival time for IL-18R knockout mice was 22 days compared to 25 days for WT mice (P<0.0001). MyD88-deficient mice had a decrease in median survival time compared to WT mice following challenge with a lethal dose of Cryptococcus, which was consistent with previously reported findings [3], [4] (**Figure 1C**). Median survival for MyD88 knockout mice was 21 days compared to 26 days for WT mice.

### The fungal burden in lungs, brains, and spleens was similar between WT and IL-1R, IL-18R, and MyD88 knockout mice

We next set up a study to assess for differences in histopathology, fungal burden, and cytokine levels between WT and knockout strains of mice. We elected to study organs at day 12 post-infection because this preceded the earliest observed mortality in this infection model, which was at day 14 post-infection in MyD88-deficient mice. We collected lungs, spleens, and brains for CFU quantification. Lungs were also analyzed for cytokines and for histopathology.

**Figure 2:**
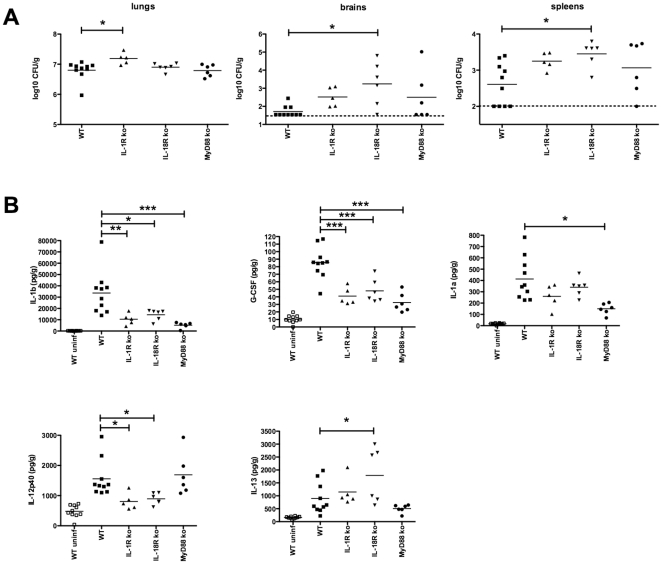
Mice were infected i.n. with 2×10^4^ *C. neoformans* organisms and euthanized 12 days post-infection. (A) Fungal burden of C57BL/6 wild-type (*n = 10*), IL-1R knockout (*n = 5*), IL-18R knockout (*n = 6*), and MyD88 knockout (*n = 6*) mice after infection with *C. neoformans*. Numbers of CFU in the lungs, brain, and spleen were determined, with results expressed as log_10_ CFU per gram of tissue and as individual data points for each animal. Bars represent the mean values and the dotted line indicates the lower limit of detection. *, *P*<0.05 and **, *P*<0.01 compared with wild-type mice. (B) Cytokine levels in the lungs of wild-type and knockout mice after infection with C. *neoformans*. Cytokines were measured by Bio-Plex assay. Lung cytokine levels were also determined in uninfected mice to establish baseline levels. Data are expressed as picograms of cytokine per gram of lung. Results are means ± SEM. *, *P*<0.05, **, *P*<0.01, and ***, *P*<0.001 compared with wild-type infected mice.

At day 12 post-infection, the CFU burden was minimally elevated in the lung homogenates from IL-1R knockout mice compared to WT mice (**Figure 2A**). This was observed despite the fact that the absence of IL-1R neither enhanced nor impaired survival following challenge with H99 at this dose. Cryptococcal levels were significantly elevated in the brains and spleens of IL-18R knockout mice compared to WT mice (**Figure 2A**). Otherwise, no differences were significant for WT versus knockout mouse CFU burdens.

### Differential induction of IL-12p40 and IL-1β was observed in lungs from IL-1R, IL-18R, and MyD88 knockout mice

We examined a total of 23 different cytokines and chemokines from lung homogenates at day 12 following i.n. infection of mice with H99 using a Bio-Rad Bio-Plex assay. Of the 23 cytokines and chemokines on the panel, 20 were significantly elevated in WT infected mice compared to WT uninfected mice (**Table 1**). On comparative analysis between infected WT mouse lungs and infected knockout mouse lungs, IL-1β levels were outstanding as these were markedly elevated in WT lungs and considerably diminished in IL-1R, IL-18R, and MyD88 knockout mice (**Figure 2B**). The only other cytokine besides IL-1β that was significantly diminished in lungs from all three knockout strains was G-CSF (**Figure 2B**). In contrast to IL-1β, IL-1α was significantly diminished only in the lungs from MyD88 knockout mice compared to WT mice. IL-12p40 levels were significantly diminished in IL-1R and IL-18R mouse lungs. Another notable finding was for IL-13, which was increased in IL-18R knockout lungs compared to WT lungs.

**Table 1:** Chemokines/cytokines increased in lungs of wild-type, IL-1R knockout, IL-18R knockout, and MyD88 knockout mice infected with *C. neoformans*.

	IL-1α	IL-1β	IL-3	IL-4	IL-5	IL-6	IL-10	IL-12p40	IL-12p70	IL-13	IL-17	eotaxin	G-CSF	GM-CSF	IFNγ	KC	MCP-1	MIP-1α	MIP-1β	TNF
Uninfected N = 10	17.0 (±6.9)	167.7 (±24.2)	0.5 (±0.1)	0.0 (±0.0)	3.8 (±0.4)	8.1 (±0.8)	31.1 (±2.8)	479.9 (±66.5)	63.3 (±3.1)	159.4 (±14.3)	24.2 (±0.8)	1557.7 (±184.7)	10.5 (±1.7)	22.3 (±1.2)	12.9 (±1.1)	33.7 (±7.6)	80.4 (±8.6)	15.2 (±3.6)	20.9 (±1.3)	144.1 (±8.5)
WT N = 10	413.0 (±59.1)	33,625.8 (±6008.1)	4.3 (±0.7)	129.4 (±12.1)	342.4 (±85.3)	91.7 (±10.1)	60.7 (±7.3)	1555.0 (±191.7)	190.7 (±28.8)	896.6 (±190.1)	48.5 (±4.6)	2915.5 (±392.5)	85.4 (±6.6)	47.2 (±5.7)	54.6 (±8.2)	314.9 (±35.7)	781.7 (±112.1)	656.0 (±115.8)	61.6 (±10.6)	277.8 (±50.0)
IL-1R KO N = 5	260.0 (±46.6)	10,484.5 (±2275.9)*	3.1 (±0.3)	152.8 (±54.6)	189.0 (±37.2)	58.2 (±16.6)	39.8 (±4.0)	806.6 (±124.4)*	120.0 (±10.8)	1146.7 (±242.3)	34.0 (±6.4)	2009.5 (±288.1)	41.0 (±5.1)*	29.0 (±2.1)*	31.2 (±4.6)	209.2 (±95.6)	1431.6 (±339.5)*	605.4 (±251.7)	42.4 (±4.8)	174.7 (±11.1)
IL-18R KO N = 6	340.5 (±32.3)	14,411.5 (±1951.8)*	3.0 (±0.2)	132.1 (±15.8)	489.9 (±81.6)	74.1 (±6.6)	51.1 (±4.5)	894.8 (±77.0)	147.4 (±11.5)	1789.4 (±434.7)*	31.8 (±5.2)	2094.3 (±327.3)	48.0 (±6.4)*	30.2 (±2.5)*	35.3 (±5.6)	197.4 (±28.7)	1152.5 (±282.9)	389.7 (±75.6)	51.1 (±2.8)	259.4 (±63.6)
MyD88 KO N = 6	149.3 (±20.0)*	5175.3 (±1021.0)*	4.4 (±1.0)	87.8 (±17.7)	150.8 (±28.3)	58.1 (±15.8)	75.7 (±12.5)	1688.9 (±281.1)	156.4 (±18.6)	504.8 (±63.8)	47.7 (±9.3)	4158.0 (±424.4)	32.5 (±5.2)*	51.2 (±6.8)	73.7 (±19.5)	154.3 (±44.0)	844.3 (±175.1)	370.1 (±78.3)	66.6 (±12.3)	264.1 (±46.3)

Cytokines were measured by Bio-Plex assay from lungs harvested 12 days post-infection. Values are expressed in pg/g lung as means ± S.E.M. All cytokines included in the table are significantly elevated in wild-type infected lungs compared to wild-type uninfected lungs (P<0.05). No significant differences were observed for IL-2, IL-9, or RANTES in wild-type infected versus wild-type uninfected lungs. *, P<0.05 for wild-type compared to knockout infected lungs.

### Histopathology showed minimal differences between lungs of WT, IL-1R, IL-18R, and MyD88 knockout mice infected with Cryptococcus

**Figure 3:**
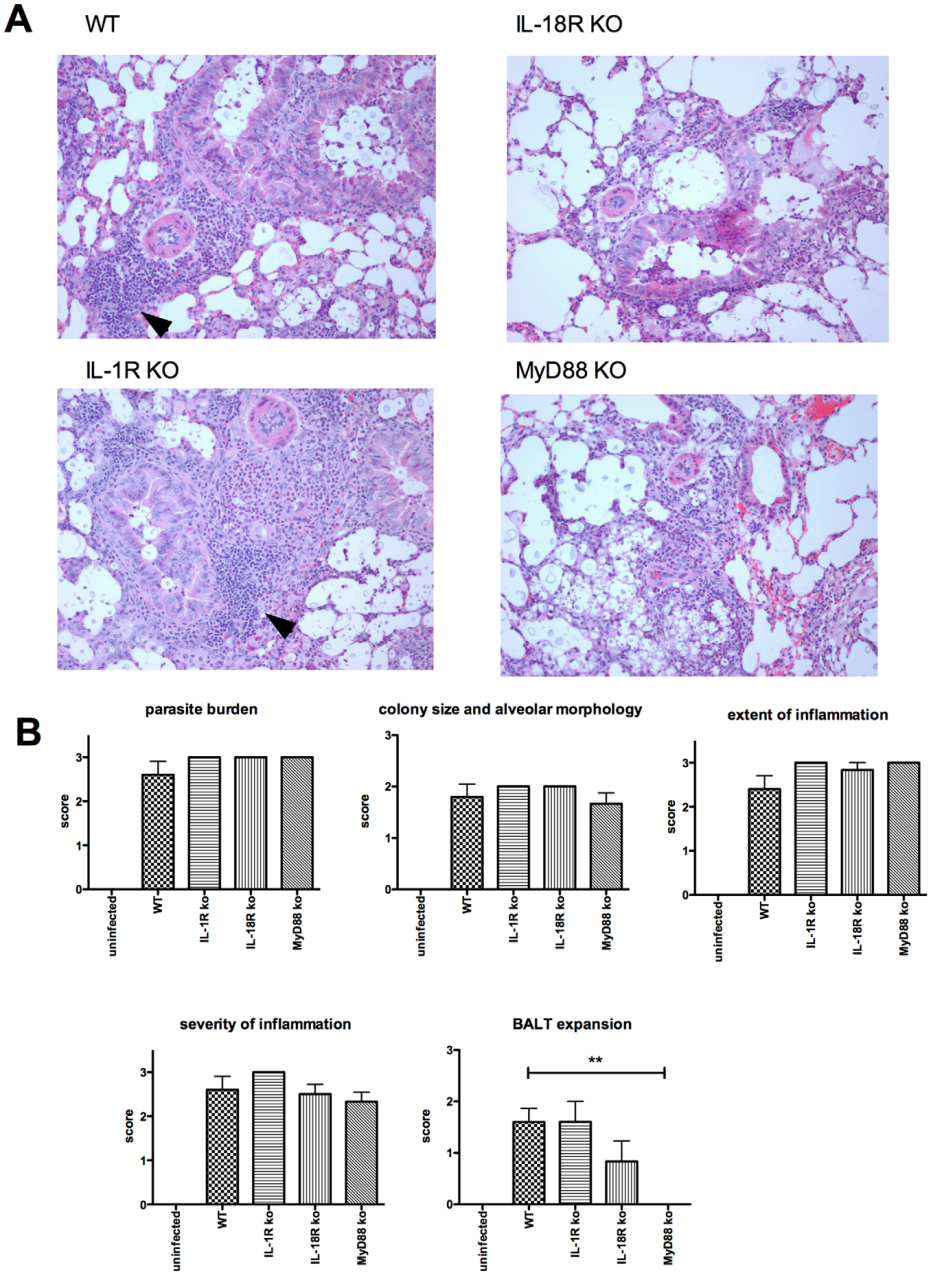
Histopathological images of lungs of wild-type, IL-1R knockout, IL-18R knockout, and MyD88 knockout mice 12 days following challenge with 2×10^4^ *C. neoformans* organisms i.n. (A) Histological samples were prepared as described in Methods. Images of H & E stained slides were taken under the light microscope at 20X power objective. Moderate to severe BALT expansion is noted in the lung sections of wild-type (*n = 10*) and IL-1R knockout mice (*n = 5*) compared to MyD88 knockout (*n = 6*) or IL-18R knockout mice (*n = 6*). The arrows point to expanded BALT in the lung sections of wild-type and IL-1R knockout mice. (B) Histopathological assessment of lungs from wild-type and knockout mice after infection. Sections were scored blind by a pathologist for the indicated observations on a scale from zero (none seen) to three (severe or maximal). **, *P*<0.01 compared with wild-type infected mice.

Lung sections from WT and knockout mice 12 days post infection were evaluated for histopathological findings (**Figure 3A**). Individual and clusters of fungal microorganisms were seen in both conducting airways and alveolar spaces in addition to interstitium. However, fungal microorganisms were more abundant in the alveolar spaces in proximity to the conducting airways. Associated inflammatory cells were composed of both acute (neutrophils and lesser number of eosinophils) and chronic inflammatory cells (histiocytes, foamy macrophages, few multinucleated giant cells, and lymphocytes). A significant increase in the number and size of lymphoid aggregates in the interstitium around bronchiolovascular bundles was noted in lung sections (see arrows in **Figure 3A**). Scattered foci of dense neutrophilic infiltrates were noted in areas containing dense populations of fungal microorganisms in all groups. No goblet cell hyperplasia was seen in the conducting airways.

Lung sections from WT and knockout mice 12 days post infection were scored for fungal burden, colony size and alveolar morphology, extent of inflammation, and the degree of bronchus-associated lymphoid tissue expansion (BALT) expansion, as previously described [3]. Scoring was performed in a blinded fashion by a pathologist on a scale from 0 to 3, with 0 being minimal, 1 mild, 2 moderate, and 3 severe. With the exception of BALT expansion, which was significantly diminished for MyD88 knockout mice, WT and knockout mice had similar pathologic findings including the extent and severity of inflammation (**Figure 3B**).

### Evaluation of TLR9 deficient mice following cryptococcal infection

**Figure 4:**
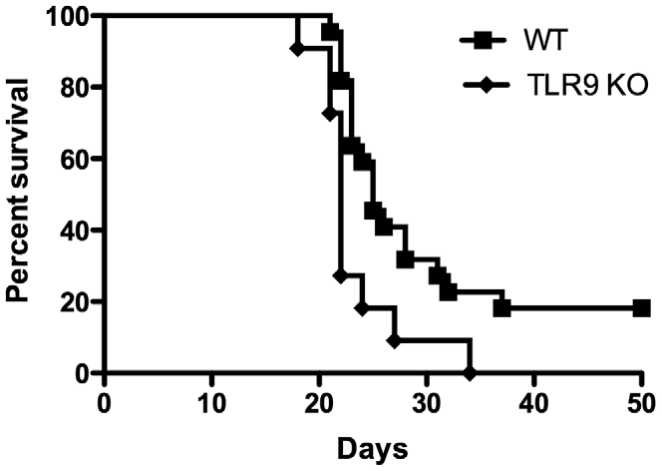
Survival of C57BL/6 wild-type and TLR9 knockout mice following infection with *C. neoformans*. Mice were infected i.n. with 2×10^4^ *C. neoformans* organisms and were monitored for signs of disease and euthanized when the signs of disease were severe. TLR9 knockout (*n = 11*) mice had a significantly different survival curve compared to wild-type mice (*n = 22*), *P*<0.01. Data are combined from two independent experiments with similar results.

**Figure 5:**
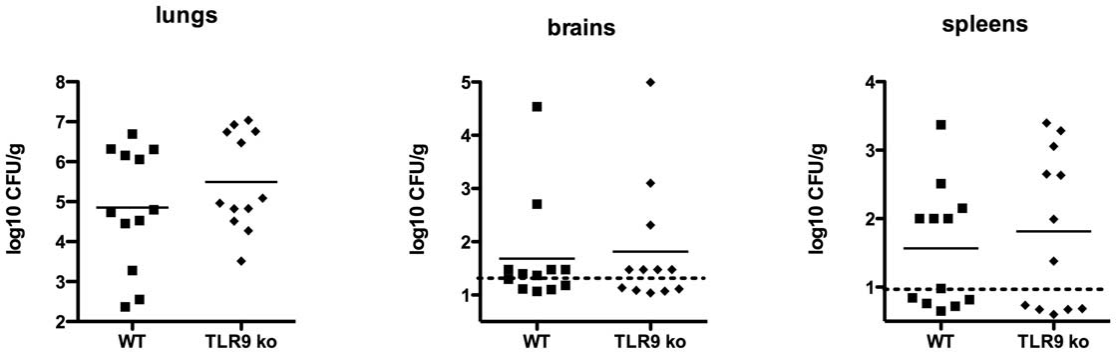
Mice were infected i.n. with 210^4^ *C. neoformans* organisms and euthanized 12 days post-infection. (A) Fungal burden of C57BL/6 wild-type (*n = 12*) and TLR9 knockout (*n = 12*) mice after infection with *C. neoformans*. The numbers of CFU in the lungs, brain, and spleen were determined. Data are expressed as log_10_ CFU per gram of tissue. The results are expressed as individual data points for each animal and the bars represent the mean values. The dotted line indicates the lower limit of detection. Data are combined from two independent experiments with similar results.

**Figure 6:**
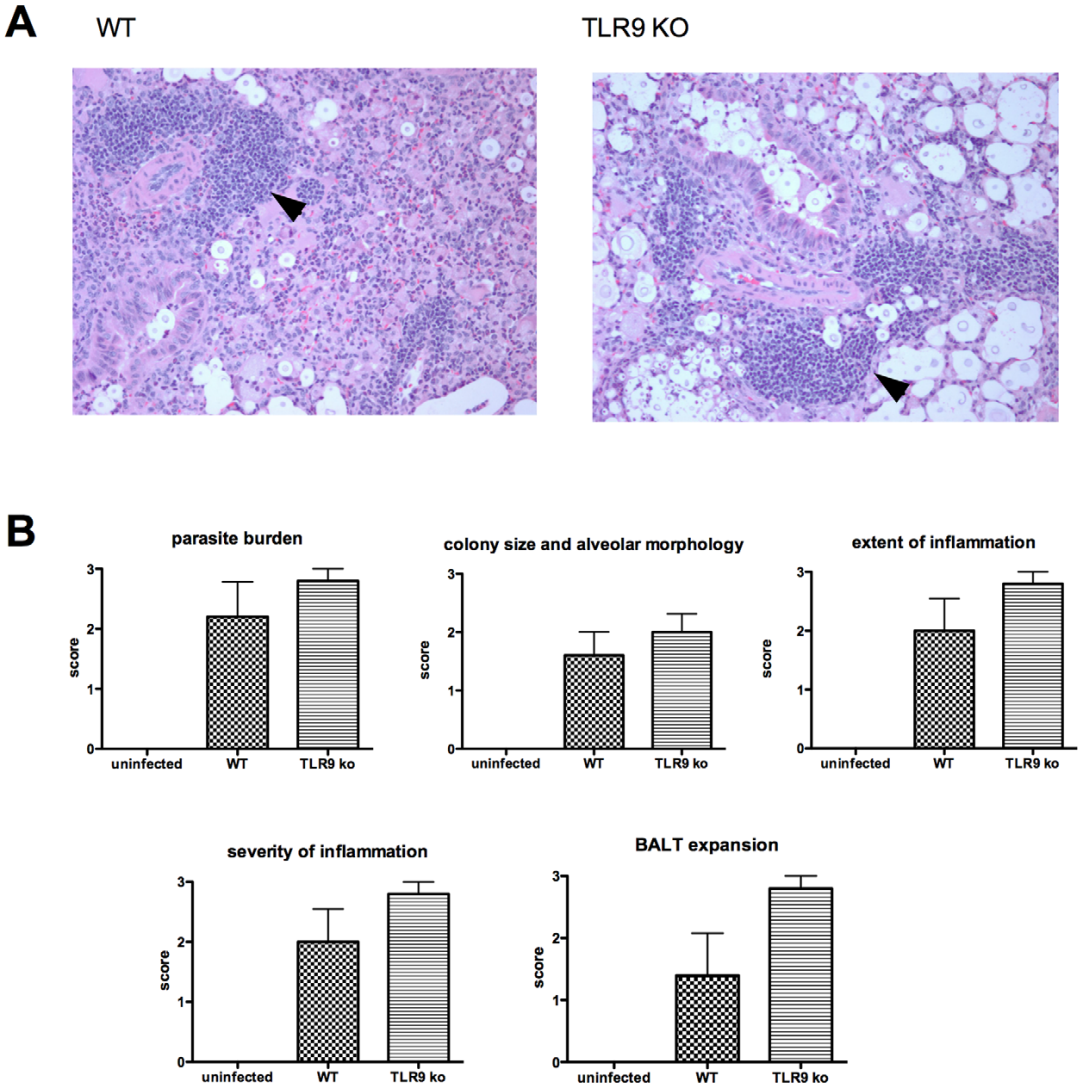
Histopathological findings in the lungs of wild-type and TLR9 knockout mice 12 days following challenge with 210^4^ *C. neoformans* organisms i.n. (A) Histological samples were prepared as described in Methods. Images of H & E stained slides were taken under the light microscope at 20X power objective. No differences were seen in lungs with respect to cryptococcal burden and inflammation between infected wild-type and TLR9 knockout mice. (B) Histopathological assessment of lungs from wild-type and knockout mice after infection (*n = 5* per group). Sections were scored blind by a pathologist for the indicated observations on a scale from zero (none seen) to three (severe or maximal). The arrows point to expanded BALT in the lung sections.

In addition to IL-1R and IL-18R, TLR9 uses the adaptor MyD88 for its signaling. Because TLR9 reportedly plays a protective role in cryptococcal infection [5], [6], we wanted to confirm its protective role in our infection model. We infected TLR9 knockout mice with 2×10^4^ CFU of H99 by i.n. inoculation and monitored for survival. TLR9-deficient mice did indeed exhibit significant differences in survival curves compared to WT mice (**Figure 4**). In two independent combined experiments, median survival was 26 days for WT mice versus 22 days for TLR9 knockout mice. In two additional independent experiments, WT (n = 12) and TLR9 knockout mice (n = 12) were infected with the same dose of H99. Mice were sacrificed at day 12 post infection and organs processed for assessment of fungal burden, cytokines, and histopathology. Overall, no significant differences were observed for CFU values for lung, brains, or spleens in WT versus TLR9 knockout animals (**Figure 5A**). Of the 23 cytokines and chemokines detected on the Bio-Plex panel, eight specific cytokines and chemokines were elevated in lungs from infected animals, but no significant differences were observed for WT versus TLR9 knockout animals (see **Table 2** **)**. Finally, no global differences in lung pathology were observed between lungs of WT and TLR9 knockout mice at day 12 following infection with H99 when the same scoring criteria were applied as for the previous experiment (**Figure 6A and B**).

**Table 2:** Chemokines/cytokines increased in lungs of wild-type and TLR9 knockout mice infected with *C. neoformans*.

	IL-1α	IL-1β	IL-4	IL-6	IL-12p40	KC	MCP-1	MIP-1α
Uninfected N = 7	77.9 (±24.4)	793 (±105)	2.6 (±1.1)	7.7 (±1.2)	330 (±86)	63.5 (±3.2)	141.8 (±27.4)	40.8 (±22.9)
WT N = 12	325.5 (±81.0)	9761 (±2869)	277.1 (±64.6)	91.4 (±30.4)	1191 (±308)	232.4 (±27.6)	801.9 (±201.7)	487.7 (±149.3)
TLR9 KO N = 12	261.6 (±82.0)	7236 (±2383)	166.9 (±29.4)	84.4 (±33.4)	785 (±177)	165.8 (±26.1)	592.7 (±130.0)	394.0 (±120.9)

Cytokines were measured by Bio-Plex assay from lungs harvested 12 days post-infection. Values are expressed in pg/g lung as means ± S.E.M. All cytokines included in the table are significantly elevated in wild-type infected lungs compared to wild-type uninfected lungs (P<0.05). No significant differences were observed between values for wild-type and TLR9 knockout lungs. Data are the combined results from two independent experiments.

## Discussion

Previous studies have shown that MyD88 and certain TLRs contribute to host protective responses in cryptococcal infection. MyD88 knockout mice had significantly reduced survival compared with WT C57BL/6 mice after pulmonary and intravenous challenge with *C. neoformans* and TLR2 knockout mice died significantly sooner following pulmonary challenge but not intravenous challenge [3], [4]. TLR4 has been shown to recognize cryptococcal capsular polysaccharide [11]. However, no strong phenotype for TLR4 has been observed in survival models using Cryptococcus [3], [4], [12]. Furthermore, the impact of TLR9 has not been demonstrated in survival studies but has been shown to influence clearance of the organism from lungs [5], [6].

Here we sought to establish the contributions of many of the receptors that utilize MyD88-dependent signaling pathways. We elected to intranasally inoculate mice with a highly virulent strain of Cryptococcus, H99, at a dose that kills WT C57BL/6 mice at an average time of 25–26 days. We confirmed that MyD88 knockout mice have reduced median survival time compared to WT mice and demonstrated that TLR9 knockout mice also have a significantly reduced median survival time compared to WT mice using this model. However, we found that neither TLR2 knockout nor TLR2/TLR4 double knockout mice exhibited differences in survival compared to WT mice using this infection model (unpublished data).

In our studies with IL-18R and IL-1R knockout mice, several observations were notable. First, IL-18R contributes to the protective response driven by MyD88, as demonstrated by the decreased median survival time in IL-18R knockout mice compared to WT mice. This corroborates with previously reported data demonstrating a protective role for IL-18 [9]. Secondly, IL-1R does not contribute to the overall survival in this infection model. Third, very high levels of IL-1β are induced in lungs of WT mice infected with H99 but IL-1β levels were markedly reduced in IL-1R, IL-18R, and MyD88 knockout mouse lungs, suggesting that expression of each of these molecules contributes substantially to IL-1β production. Nevertheless, diminished levels of pulmonary IL-1β do not necessarily correlate with survival outcomes, as evidenced by the similar survival curves for WT and IL-1R knockout mice. Also, while TLR9 knockout mice had a survival disadvantage, the levels of IL-1β were equal to those of WT mice.

Modestly diminished levels of IL-12p40 were observed in the lungs from IL-1R and IL-18R knockout mice at day 12 post-infection, suggesting that a reduced Th1 response contributed to the decreased survival of these knockouts during infection. The resolution of pulmonary *C. neoformans* infection in experimental murine models has been associated with the induction of Th1-type cytokine responses [13]. The cytokine IL-12p40, which is a subunit for both IL-12 and IL-23, plays a critical role in host defense through the induction of IFNγ and subsequent development of Th1 cells [14]. In addition, IL-23 is important for the promotion of IL-17 production and driving Th17 responses, which participate in host immune responses following cryptococcal infection [15], [16], [17]. A cooperative relationship between IL-18 and IL-12p40 has been described in Cryptococcus infection [9], [10], [18], so the diminished levels of IL-12p40 in IL-18R knockout mice are not surprising. Likewise, levels of the Th17 cytokine IL-17 were low but detectable in infected lungs at day 12. There was a trend, albeit not significant, towards diminished IL-17 levels for both IL-1R-deficient and IL-18R-deficient infected mice in comparison to wild-type infected mice (see **Table 1**). IL-17 might peak in lungs at times other than day 12 post-infection, so the possibility exists that greater differences could be observed between wild-type and knockout mice.

We also found that IL-18R deficient mouse lungs have significantly elevated levels of IL-13 at day 12 following infection. IL-13 is cytokine produced by activated CD4+ lymphocytes, so the increase may reflect a shift towards a Th2 response, which has been associated with increased mortality [19]. Other cytokines and chemokines associated with Th2 responses (e.g., IL-4, IL-5, and IL-10) did not differ between wild-type and knockout mouse lungs at day 12 in the experiments described here. However, the levels of these cytokines and chemokines were quite low and the possibility that they might peak in the lungs at a time other than day 12 post-infection cannot be excluded.

*C. neoformans* strain H99 is highly virulent in mouse models with central nervous system dissemination observed relatively early following pulmonary challenge. Perhaps because H99 is so virulent, significant differences in survival between mouse strains challenged with *C. neoformans* have been generally difficult to demonstrate. Giles et al. investigated the role of surfactant protein A (SP-A), a putative mediator of host defense against Cryptococcus [20]. While they were able to demonstrate that SP-A binds to Cryptococcus, SP-A-deficient and wild-type mice infected via intranasal inoculation with *C. neoformans* H99 demonstrated no differences in lung CFU nor overall susceptibility to infection. In a similar infection model, McQuiston et al. assessed the role of sphingosine kinase 1 (SK1) in the host response to Cryptococcus infection [21]. Following intranasal challenge of C57BL/6 wild-type mice and SK1 knockout mice with *C. neoformans* H99, no significant differences in either survival or fungal burden in brain, liver, or spleen were observed between the two groups. Finally, Zhang et al. studied the impact of IL-4/IL-13 deletion on murine cryptococcosis [17]. They compared wild-type Balb/C mice versus IL-4/IL-13 double knockout mice challenged intratracheally with *C. neoformans* H99. While fungal burden was significantly diminished in the lungs of knockout mice, deletion of IL-4/IL-13 was insufficient to prevent CNS dissemination and the overall survival of the knockout mice was not statistically significant in comparison to wild-type mice. Given this context, the survival differences described in our study, while not dramatic, are nevertheless impressive.

The inflammatory response can be deleterious in cryptococcosis. Cryptococcosis is frequently associated with the immune reconstitution inflammatory syndrome (IRIS) in patients with AIDS on antiretroviral therapy [22]. In the murine infection model used for our studies, considerable evidence of inflammation was observed through lung histopathology and cytokine levels, but enhanced or diminished inflammation did not appear to correlate with survival with any of the knockout strains of mice tested. The host inflammatory response may not directly correspond with a significant decrease in survival. Survival might instead correlate with dissemination outside the lung and the subsequent development of meningoencephalitis.

One observation was the paucity of BALT in lungs from MyD88 knockout mice infected with Cryptococcus. The presence of BALT is indicative of an immune response in mice but is not specific as it has been associated with either CD4 or CD8 cell recruitment in rodent models of infection [23], [24]. The significance of this isolated finding in our study is unclear. In our previous study, we did not observe any specific differences in inflammatory responses between WT and MyD88-deficient mice infected with *C. neoformans*. However, in that study, we used a different strain (serotype A 145) and examined lungs at a different time point following infection [3].

In our infection model, no single cytokine specifically correlated with survival advantages or disadvantages. This emphasizes the complexity of signaling, cytokine and chemokine production, and host inflammatory cell recruitment and response during cryptococcal infection. The differential pattern of cytokine production in WT versus knockout mouse lungs following cryptococcal infection may be reflective of different types of cells that are recruited to the lungs.

Multiple factors impact survival following intranasal delivery of this dose of H99, including a combination of central nervous system disease and pulmonary disease. Our experimental findings suggest that IL-18R and TLR9 significantly contribute to MyD88-dependent host defense in cryptococcal infection. Further studies on the processing of the active form of IL-18 during cryptococcal activation are warranted.

## Materials and Methods

### Ethics statement

Experimental protocols involving animals were approved by the University of Massachusetts Medical School Institutional Animal Care and Use Committee. Mice were euthanized if they exhibited severe clinical signs of disease including listlessness, hunched posture, poor body condition, difficulty ambulating including ataxia, rough hair coat, failure to groom, increased respiratory rate, and difficulty breathing.

### Mice

IL-1R knockout and IL-18R knockout mice on the C57BL/6 background were purchased from the Jackson Laboratory (Bar Harbor, ME) and bred at the University of Massachusetts Medical School. MyD88 knockout, TLR9 knockout, TLR2 knockout, and TLR2/TLR4 double knockout mice were obtained as a gift from Dr S. Akira (Osaka University, Japan). All WT control C57BL/6 mice were age-matched and were purchased from Jackson Laboratory. Single nucleotide polymorphism-based genome scanning analysis of all knockout strains confirmed that the C57BL/6 background was ≥94% (Jackson Laboratory).

Mice were between 8 and 12 weeks old upon infection. Mice were bred and housed in the animal facility at the University of Massachusetts Medical School.

### Inoculation of mice

*C. neoformans* strain H99 [25], obtained from J. Heitman (Duke University Medical Center), was used in all experiments. Pulmonary *C. neoformans* infections were initiated by intranasal delivery as previously described (8, 9). Briefly, mice were anesthetized with 2% isoflurane and then given a yeast inoculum of 2×10^4^ colony forming units (CFU) of *C. neoformans* strain H99 in 30 µl of sterile PBS pipetted directly into the nares. The inocula used were verified by quantitative culture on Sabouraud-dextrose agar.

### Preparation of mouse organs

In certain experiments, mice were sacrificed at day 12 post-infection. Mice were fully anesthetized with isoflurane, then exsanguinated by cardiac puncture. The lungs were removed and the left lung was fixed in 10% buffered formaldehyde for 24 h. One half of the right lung, the brain, and spleen were each homogenized in 0.5 ml of sterile saline and aliquots were diluted and plated onto Sabouraud-dextrose agar plates. The remaining half of the right lung was stored at −80°C until cytokine analysis was performed.

### Cytokines

Frozen lungs were thawed on ice and homogenized in 0.2 ml of PBS. Homogenates were spun down at 20,000 g for 10 min at 4°C. Supernatants from lung homogenates were analyzed using the Bio-Plex system and a Luminex 100™ analyzer (Bio-Rad) according to the manufacturer's instructions. Results are expressed as points for each mouse sample in picograms and are normalized to the organ weight.

### Pathology

Fixed tissue samples were embedded in paraffin for sectioning. Organs were processed for hematoxylin and eosin (H&E) staining using standard protocols. Cross sections were observed by light microscopy. Sections were read in a blinded fashion by a pathologist (A. Akalin) and scored for the degree of fungal infiltration and inflammation.

### Statistical Analysis

All analyses were performed using GraphPad Prism. Survival data was analyzed using the log-rank test. Data for samples from wild-type, IL-1R knockout, IL-18R knockout, and MyD88 knockout mice were analyzed by one way ANOVA with Dunnett's multiple comparison test. Statistical comparisons were made for samples from wild-type and TLR9 knockout mice utilizing the Student's *t* test. *P* values of <0.05 were considered significant. Statistical calculations involving CFU were performed on log_10_-transformed values.
